# Self-Directed Learning Strategies: A Comparative Evaluation of Facilitated and Self-Paced Methods

**DOI:** 10.7759/cureus.88982

**Published:** 2025-07-29

**Authors:** Meghana Padwal, Lalna Takale, Asmita Phadke, Gayatri Gawade Maindad, Mandar Karmarkar, Aarati B Pokale

**Affiliations:** 1 Biochemistry, Bharati Vidyapeeth (Deemed to be University) Medical College, Pune, IND; 2 Physiology, Bharati Vidyapeeth (Deemed to be University) Medical College, Pune, IND; 3 Forensic Medicine, Bharati Vidyapeeth (Deemed to be University) Medical College, Pune, IND; 4 Community Medicine, Bharati Vidyapeeth (Deemed to be University) Medical College, Pune, IND

**Keywords:** cbme, facilitated, medical education, self-directed learning, self-paced

## Abstract

Background: Self-directed learning (SDL) is a process in which the learner takes initiative, defines learning objectives, and achieves the learning outcome. One of the roles of an Indian medical graduate (IMG) as per the competency-based medical education (CBME) curriculum, is to become a lifelong learner, which can be achieved by SDL. The strategies used for the implementation of SDL may be facilitated, in which the faculty facilitates learning, and self-paced SDL is without a facilitator. The studies using either one of these strategies have been done so far. Hence, this study was conducted to compare the effectiveness of both strategies and evaluate their impact on learning outcomes.

Objective: To compare the effectiveness of facilitated and self-paced learning as two strategies of SDL.

Materials and methods: A prospective, interventional study was conducted in the Department of Biochemistry and included 119 students in the first year of MBBS in a medical college attached to a tertiary care teaching hospital in Pune. The department faculty were sensitized about SDL, and the topic for SDL was finalized. The first session of SDL was sensitizing the students about SDL and conducting a validated multiple-choice question-based pre-test on the topic of enzyme inhibition and clinical enzymology. The students were divided into four batches by convenience sampling, out of which two batches were allotted to the facilitated SDL group and two batches to the self-paced SDL group. There was a 14-day intersession period during which learning resources were shared and discussions were conducted in the facilitated group. In the second session following this period, a post-test was conducted, and a debriefing was done to cover the learning gap. Feedback using an online form was taken from both students and faculty.

Results: There was improvement in marks obtained in pre- and post-tests in both facilitated (21.27 and 52.55) and self-paced (21.07 and 45.36) SDL groups, which was statistically significant (p<0.001). There was a difference in the post-test marks of the facilitated (52.55) and self-paced groups (45.36), which was statistically significant (p<0.001).

Conclusion: The lifelong learning skill can be fostered by incorporating SDL in their curriculum. The SDL module needs to be carefully chosen and designed as per training needs, resources available, and learners’ profiles. The CBME biochemistry curriculum has a lot of scope for covering some part of the syllabus by SDL, and also includes core competencies in the future. SDL can be undertaken using facilitated and self-paced strategies, which promote self-motivation, accountability, and critical thinking to achieve learning objectives. Thus, methodical SDL conducted using these strategies will pave the way for students to be lifelong learners by incorporating it into their curriculum.

## Introduction

Knowles has defined self-directed learning (SDL) as "a process in which individuals take the initiative, with or without the help of others, in diagnosing their learning needs, formulating learning goals, identifying human and material resources for learning, choosing and implementing appropriate learning strategies, and evaluating learning outcomes" [1]. Additionally, the learning style of each learner is also crucial in the learning process [2].

The competency-based medical education (CBME) regulations of 2023, dated August 1, 2023, mention that one of the goals of an Indian medical graduate (IMG) is to be a lifelong learner who is committed to continuous improvement of skills and knowledge. As per the regulations, specific teaching hours for each subject have been allotted to SDL. Thus, it is an inevitable part of their curriculum [3].

The traditional medical education was teacher-centered, whereas the CBME curriculum promotes student-centered learning. The students should be actively involved in the learning process, and the role of the teacher should be that of a facilitator. SDL is one of the learning methods that facilitates active learning and critical thinking [4]. In medicine, newer frontiers of diagnosis and management are a reality, and to keep up, IMG needs to continuously update itself. SDL provides a way and means of becoming a lifelong learner, and it is an important aspect of adult learning [5].

The process of implementation of SDL involves planning SDL sessions, conducting an assessment, and giving feedback. Planning the SDL session includes faculty training, if required, and selection of the topic. The SDL can be conducted in three sessions. Session one is an orientation to the concept of SDL, discussing learning objectives for the selected topic and resources that can be used. This is followed by an intersession period of seven to 10 days during which the students prepare the topic. The second session is conducted to summarize or debrief the topic, assess SDL, and provide feedback about the learning experience [1].

SDL can be conducted broadly by two methods, facilitated and self-paced. Facilitated SDL involves guidance or facilitation from faculty to achieve the learning objectives. Self-paced SDL, on the other hand, does not involve faculty to facilitate learning [6].

The literature available emphasizes SDL for lifelong learning and has been implemented in various ways. A comparative study using facilitated and self-paced SDL as two strategies is less explored and is an area of interest to be navigated. Hence, the present study was conducted to evaluate facilitated and self-paced learning as two strategies of SDL. 

## Materials and methods

This was a prospective interventional study carried out at Bharati Vidyapeeth (Deemed to be University) Medical College, Pune. Before commencement, Institutional Ethics Committee approval (Ref. BVDUMC/IEC/02, dated 20/09/2023) was obtained. Informed consent was sought from the participants.

Study details

The study population included first-year MBBS students of the 2023-24 batch. Students who were present for both the pre-test and post-test were included in the study. Students who did not give consent to take part in the study were excluded. A total of 119 students participated in the study. The study was conducted from September 2023 to December 2023.

Experimental protocol

The study was conducted in four steps as follows:

Planning of the Self-Directed Learning Session

Enzyme inhibition and clinical enzymology were the topics selected for SDL. The learning objectives were outlined, and faculty were sensitized and trained in the conduct of SDL. Pre-test and post-test questionnaires were prepared and validated by the faculty members before the actual conduct of SDL (Appendix 1).

First Session of Self-Directed Learning (60 Minutes Duration)

Students were oriented to the concept of SDL and both its types: facilitated and self-paced. Topics selected for SDL were shared with them. A pre-test consisting of 35 multiple-choice questions (MCQs), which were both recall- and scenario-based, was administered to all the students using Microsoft Forms (Microsoft Corporation, Redmond, Washington, United States). The students were divided into four batches by convenience sampling, out of which two batches were allotted to the facilitated SDL group (A: 60 students) and two batches to the self-paced SDL group (B: 59 students). For ease of interaction, the facilitated SDL batch was further divided into three small batches. These small batches were guided by three faculty members as mentors. Each mentor created a WhatsApp (WA) (Meta Platforms, Inc., Menlo Park, California, United States) group with their respective small batch. The self-paced SDL batch was not assigned any mentor and was advised to explore resources like reference books, YouTube videos (Google LLC, Mountain View, California, United States), etc., in order to achieve the learning objectives.

Intersession Period of 14 days

During this period, the students prepared the topic by exploring resources like textbooks, YouTube videos, etc. For the facilitated SDL group, mentors also provided learning resources like short videos and reels of clinical scenarios and triggered learning by posing questions to the group from time to time. Mentors addressed students’ queries and steered the discussion in the right direction.

In the self-paced SDL group, there were no such WA groups and no facilitator, and the students studied the topic on their own.

Second Session Self-Directed Learning (120 Minutes)

Post-test was conducted for both groups using the same pre-test questionnaire. A debrief session was conducted for all the students to ensure that there was no learning gap in both groups. Structured feedback using Microsoft Forms was obtained from both faculty and students to understand their perception of SDL.

Statistical analysis

Data obtained from online forms in Microsoft Excel was analyzed using IBM SPSS Statistics for Windows, Version 29 (Released 2022; IBM Corp., Armonk, New York, United States). The data was analyzed for quantitative variables by using a paired t-test. Reliability for questions of feedback was checked by Cronbach's alpha, which was 0.883 for students’ feedback and 0.703 for faculty feedback. 

## Results

The study included 119 students of the first year of MBBS, and data for pre-test and post-test scores obtained by facilitated and self-paced groups were analyzed. 

There was a statistically significant difference in the 11 pre-test and post-test scores of the facilitated SDL and self-paced group (Tables 1, 2). 

**Table 1:** Pre-test scores and post-test scores of facilitated group (n=60)

Group	N	Mean marks obtained	Paired t-test	p-value
Pre-test score	60	21.27	-21.751758	<0.001
Post-test score	60	52.55		

**Table 2:** Pre-test scores and post-test scores of self-paced group (n=59)

Group	N	Mean marks obtained	Paired t-test	p-value
					Pre-test score	59	21.07	-13.1748	<0.001
Post-test score	59	45.36							

The pre-test scores of both groups were comparable (21.27 and 21.07). The post-test scores of the facilitated and self-paced SDL groups (52.55 and 45.36) revealed improvement as compared to their pre-test scores. The mean gain in post-test scores of the facilitated group (31.29 ± 11.14) was higher than compared of the self-paced (24.30 ± 14.16) SDL group, which was also statistically significant (p = 0.003 by independent t-test).

**Figure 1:**
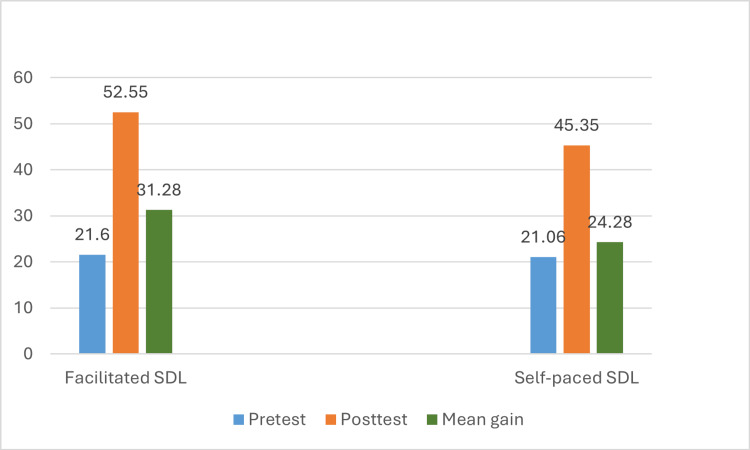
Comparison of pre-test and post-test scores and mean gain score of facilitated and self-paced groups

Thus, facilitated SDL was more effective than self-paced SDL. The difference in pre-test and post-test scores and the mean gain of both facilitated and self-paced groups is represented in Figure 1.

In online feedback, the majority of students perceived that SDL helps in improving communication skills, peer learning, and learning from online resources. The feedback also revealed that 58% of students agreed SDL is an effective teaching-learning method, 51% of students felt it improves critical thinking, 59% agreed that they would prefer SDL for future classes, and 60% of students agreed that it helps to improve learning skills. Although some students identified various challenges faced during SDL as being stressful and time-consuming. The facilitated SDL group mentioned the availability of faculty for guidance as a favorable factor for SDL. Surprisingly, the response to which group they would prefer to be in revealed almost equal votes for both the facilitated and self-paced groups. Faculty appreciated the opportunity to conduct SDL sessions. The faculty agreed (77.8%) that SDL increases the responsibility and accountability of students. The medical education curriculum is becoming student-centric, which was agreed upon by 77.8% of the faculty. Faculty also gave feedback that SDL encourages lifelong learning skills, improves academic performance, and improves team learning and peer teaching.

## Discussion

SDL was mentioned in the 1997 Medical Council of India regulations and in the CBME curriculum and was allotted specific teaching hours across all phases of MBBS [3].

SDL was defined by Malcom Knowles, and he identified five cognitive activities that need to be undertaken during SDL process (diagnose one’s own learning needs, formulate goals, identify resources for learning, choose and implement appropriate learning strategies, and evaluate learning outcomes) [7]. SDL was later expanded by Sargeant et al, emphasizing the willingness of learners to drive their own learning [8]. This highlights the autonomous student-led module of SDL [9]. 

SDL can be conducted by various methods, e.g., audio-video lectures, small group discussions, donut rounds, self-paced learning, flipped classrooms, and group projects [6]. But the essence of successful SDL is the capacity building of learners to hone critical thinking, self-management skills, social skills, communication skills, analytical skills, and research skills. So, learners with good SDL skills will be able to independently find resources about the things they wish to learn, build new concepts based on their previous knowledge, check for understanding, get the difficulties addressed, and apply learning in a practical context [7]. 

In our study, two methods of SDL, viz., facilitated SDL and self-paced SDL, were compared. We found facilitated SDL to be more effective than SDL. This finding may be due to the support by the teachers to the students in the form of sharing various online and offline resources, face-to-face interactions, and providing academic triggers during the intersession period to the facilitated group, whereas the self-paced group did not receive faculty guidance.

SDL has been conducted using various methods by the researchers. Thota S. et al. evaluated the outcome of SDL by supplementing it with a lecture and found a statistically significant increase between pre- and post-test scores in the SDL group [10]. Kollathody S et al. conducted a quasi-experimental study to compare interactive lectures and SDL. They found improvement in both groups [5]. Satyanarayan et al. compared online SDL with didactic lectures and found a significant difference between pre- and post-test scores in the SDL group [11]. Murugaiyan S et al. used problem-based learning as a method for SDL and noted its effectiveness in low, average, and high achiever students [12]. 

In our study, we obtained students’ feedback about SDL, which was very positive. Similar findings were observed by authors Patra S et al. [13]. They have reported that students enjoyed SDL sessions and found them to be an effective as well as innovative way of learning. The students also expressed that the active involvement of facilitators could impart knowledge and skills in a better manner. Other researchers have also noted students’ perceptions about SDL as promoting communication skills and critical thinking [5,14].

Thus, for the successful conduct of SDL, the responsibility of teachers is that they need to exhibit flexibility and creativity in designing assignments for students to work individually or collaboratively. To help learners become self-directed, responsibility for learning must gradually be shifted from the teacher to the student, promoting a student-centered curriculum.

 At the same time, one must remember the challenges while practicing SDL, like a high teacher-student ratio, the availability of online resources for students in the institute, and the need for more time.

To sum up, the quality of being a lifelong learner is essential for a successful career in medicine. As medical teachers, we must utilize the opportunities right from the foundation course till the final year to mold the naïve student into a self-directed learner.

Limitations

Possible resource sharing from facilitated learning group students to self-paced group students.

## Conclusions

The CBME biochemistry curriculum has a lot of scope to cover some parts of the syllabus by SDL. Faculty should carefully choose and design the module as per training needs, resources available, and learners’ profiles. In the present study, the authors felt that core competencies can also be covered by SDL. In our study, we compared the effectiveness of two strategies of SDL, facilitated and self-paced, and found that facilitated SDL was more effective as compared to self-paced SDL. The activity was appreciated by students and faculty. Methodical SDL conduct will pave the way for an IMG to be a lifelong learner. 

## Figures and Tables

**Figure 2:**
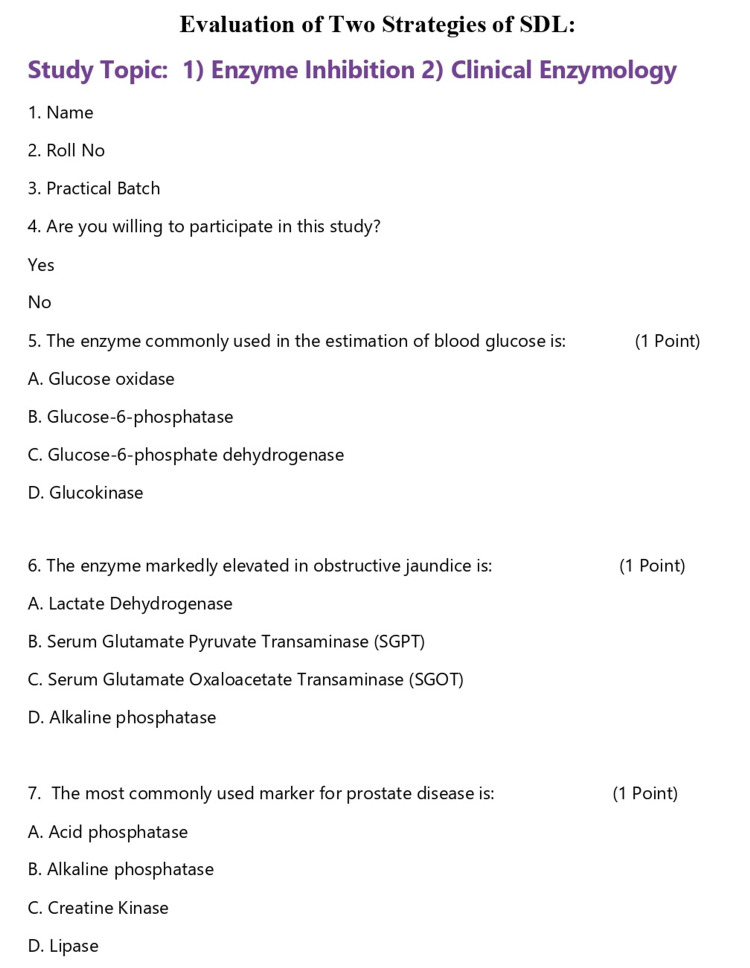
Questionnaire (part 1)

**Figure 3:**
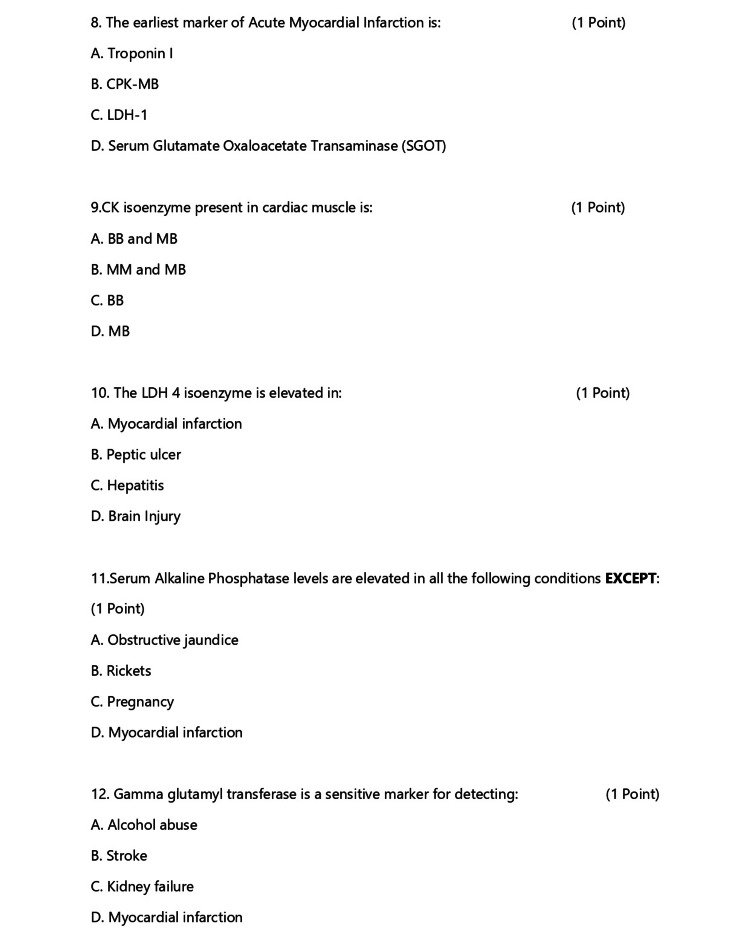
Questionnaire (part 2)

**Figure 4:**
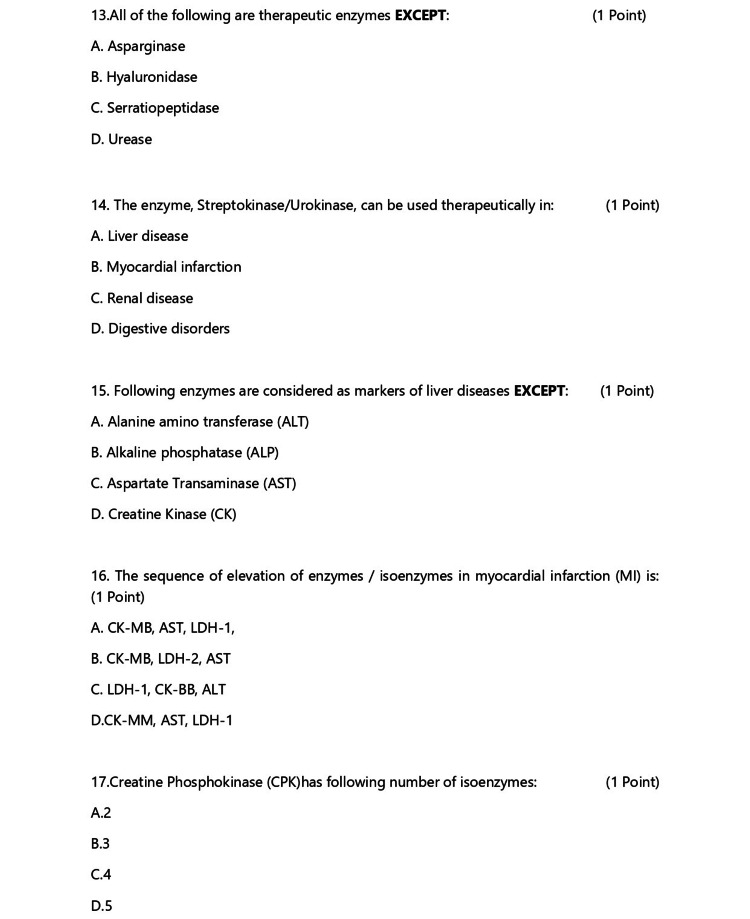
Questionnaire (part 3)

**Figure 5:**
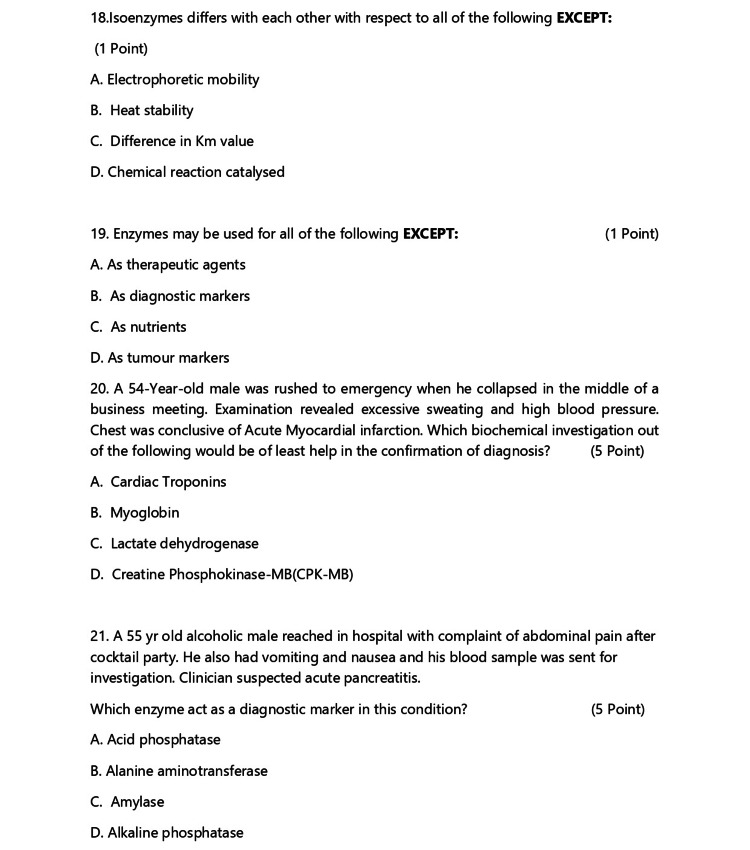
Questionnaire (part 4)

**Figure 6:**
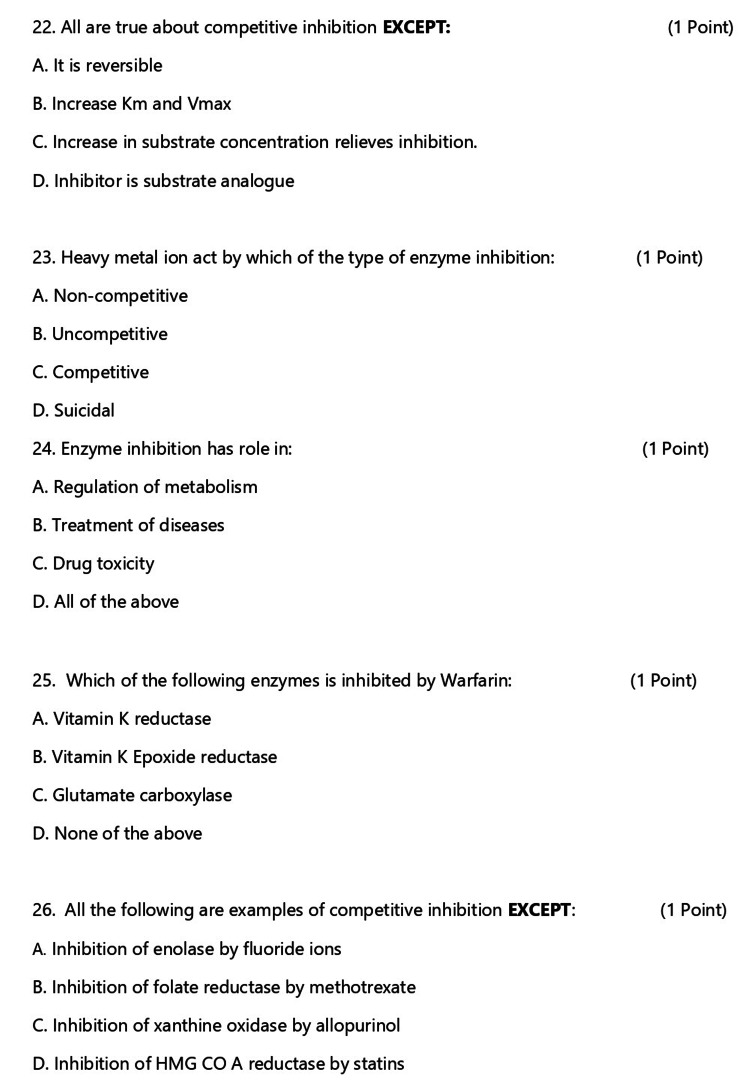
Questionnaire (part 5)

**Figure 7:**
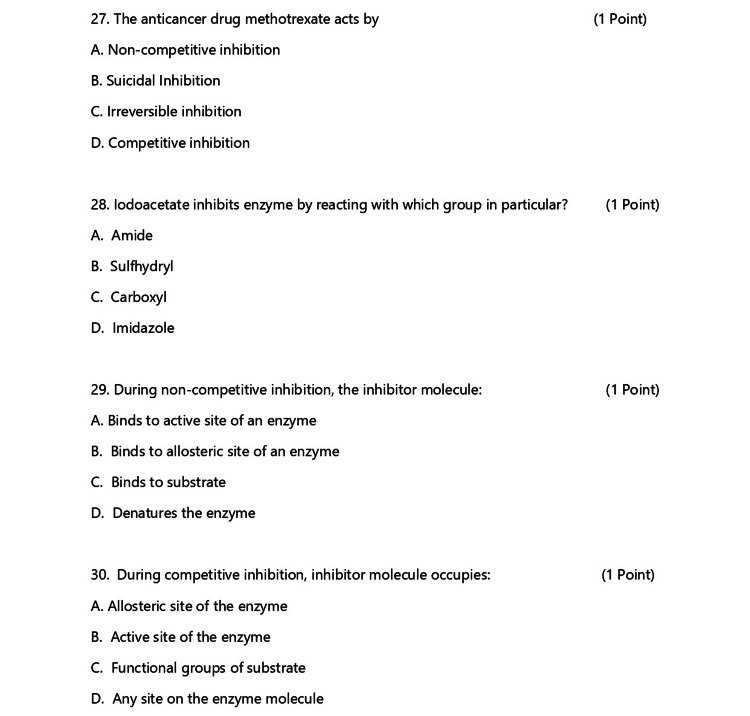
Questionnaire (part 6)

**Figure 8:**
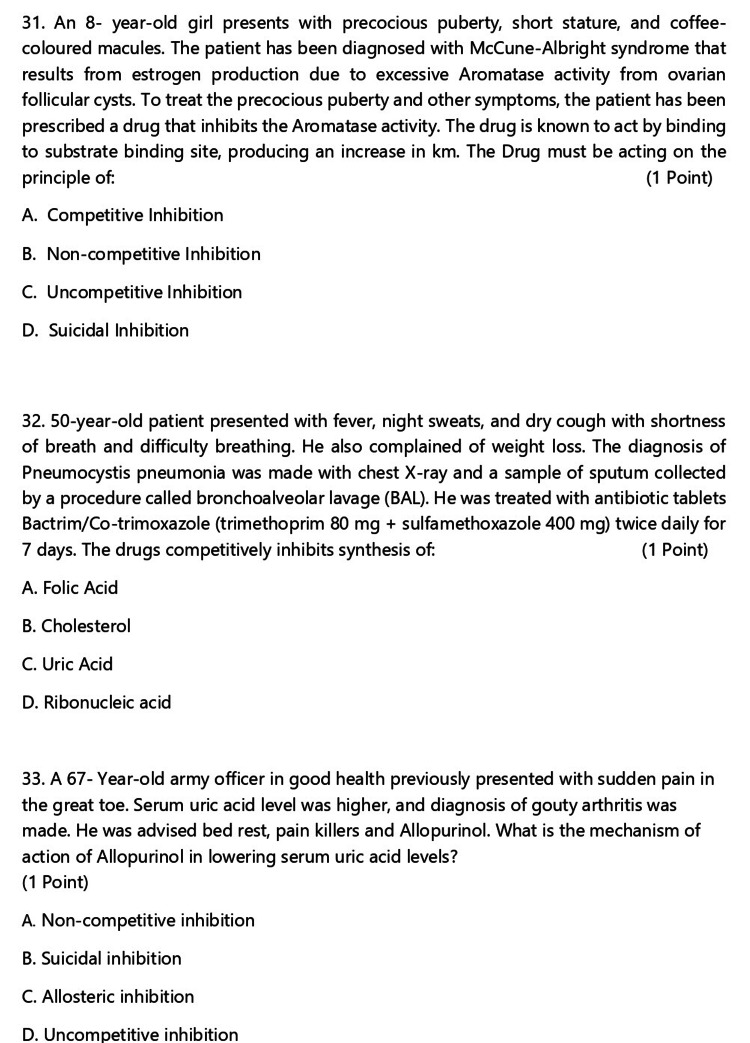
Questionnaire (part 7)

**Figure 9:**
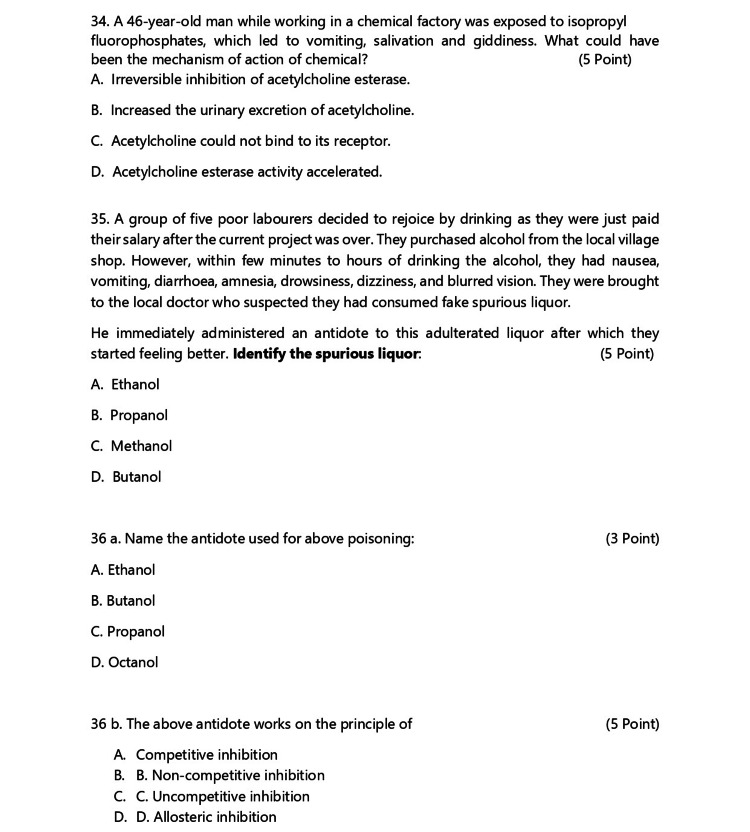
Questionnaire (part 8)

**Figure 10:**
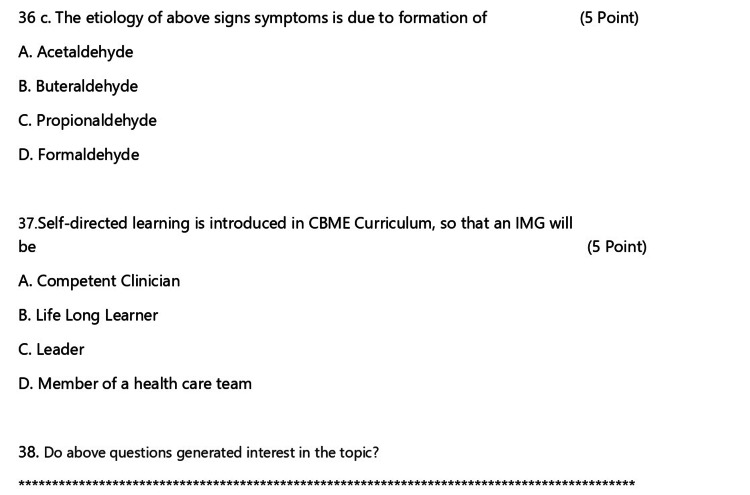
Questionnaire (part 9)

## Appendices

Appendix 1
